# Deciphering the epigenetic role of KDM4A in pancreatic β-like cell differentiation from iPSCs

**DOI:** 10.3389/fendo.2025.1697097

**Published:** 2025-10-31

**Authors:** Felipe Arroyave, Lina Méndez-Castillo, Fernando Lizcano

**Affiliations:** ^1^ Center of Biomedical Investigation, (CIBUS). Universidad de La Sabana, Chía, Colombia; ^2^ Doctoral Program in Bioscience. Universidad de la Sabana, Chía, Colombia; ^3^ Clínica, Universidad de La Sabana, Chía, Colombia

**Keywords:** induced pluripotent stem cells, knock-down, KDM4A, pancreatic β-like cells, differentiation, insulin-secreting cells

## Abstract

**Introduction:**

Pancreatic β cells derived from human induced pluripotent stem cells (hiPSCs) represent a promising therapeutic avenue in regenerative medicine for diabetes treatment. However, current differentiation protocols lack the specificity and efficiency required to reliably produce fully functional β cells, limiting their clinical applicability. Epigenetic barriers, such as histone modifications, may hinder proper differentiation and the acquisition of essential maturation markers in these cells.

**Methods:**

hiPSCs were cultured under feeder-free conditions and subjected to lentiviral transduction with shRNA constructs to silence *KDM4A*. Differentiation into pancreatic β-like cells was performed using stepwise protocols, with or without doxycycline supplementation, to evaluate the effect of *KDM4A* suppression. Gene expression was quantified by RT-qPCR, protein expression was assessed by western blotting and immunofluorescence, and functional insulin release was determined by glucose-stimulated insulin secretion (GSIS) assays. Statistical analysis was conducted using unpaired two-tailed Student’s t-tests, with significance set at *p* < 0.05.

**Results:**

A reduction in pancreatic development proteins was observed in the different differentiation states evaluated, after blocking KDM4A expression. Knockdown of KDM4A significantly reduced the expression of pancreatic β-cell genes, such as PDX1, Nkx6.1, and Ins, by 50% compared to WT iPSCs differentiated under the same conditions. Similarly, glucose-stimulated insulin secretion was reduced by approximately 80% in KDM4A-deficient β-like cells.

**Conclusions:**

These results emphasize the critical role of histone demethylation in hiPSC differentiation toward β cells. Our findings identify KDM4A as a key epigenetic regulator, suggesting that its modulation could enhance the generation of functional β cells for regenerative medicine in diabetes.

## 1 Introduction

Epigenetic control through histone modifications is a critical determinant of cell fate during development and reprogramming. (1–5). During endocrine differentiation of human embryonic stem cells, dynamic resolution of bivalent chromatin states (H3K4me3/H3K27me3) and removal of Polycomb-mediated repression are essential for the activation of key developmental regulators (6, 7).

Additionally, repressive chromatin marks, especially the trimethylation of lysine 9 of histone 3, (H3K9me3) reduce the expression of key genes in embryonic development (8). KDM4A, a histone demethylase targeting H3K9me3, has been associated with chromatin remodeling in stem cells, yet its role in endocrine pancreas specification is not well defined. KDM4A is associated in the regulation of genes related with cell proliferation, genome stability, and the preservation of pluripotency in maternal stem cells (9–16). Knockdown of KDM4A (KD-KDM4A) in cellular models has been shown to reduce cell proliferation, making it a subject of interest in various cancers (14, 17).

Differentiation of iPSCs into pancreatic β-cells remains suboptimal, because a fall to produce β-cells completely mature. Among of the difficulties that immature β-cells have is low energy efficiency, lack of identity and low capacity to respond to cellular stress. (7, 18–20). The heterochromatic mark H3K9me3 might poses a barrier to activation of β-cell enhancers pattern that would affect genes that harmonically regulate insulin secretion. A critical aspect of demethylase activity was demonstrated in cloning experiments using non-human primates, where an epigenetic barrier had to be overcome in experiments involving SCNT (somatic cell nuclear transfer). Tarrier was characterized by hypermethylation of H3K9me3, which prevented embryo development and was only overcome with histone demethylase family 4 (KDM4), which established the first cloned primates. (7, 8, 21–26).

We propose that KDM4A is essential for effective β-cell differentiation by enabling the activation of lineage-specific transcriptional programs. Using KDM4A knockdown iPSCs, we test whether impaired H3K9me3 clearance disrupts endocrine specification and β-cell maturation, offering insights into the epigenetic barriers that limit the generation of functional cells for diabetes therapy.

## 2 Materials and methods

### 2.1 Material

The 20b iPSCs were acquired from the Harvard Stem Cell Institute (HSCI) (Boston, United States) and were maintained on vitronectin in mTeSR1™. The medium was changed daily, and the cells were passaged when they reached 85–90% confluency. Dulbecco´s phosphate-buffered saline w/o calcium and magnesium (DPBS; 14190250), L-Glutamine (25030081), heat-stable recombinant human basic bFGF (100-18BHS-100UG), heat-stable FGF10 (PHG0375), B27 supplement minus vitamin A (12587010), human Noggin recombinant protein (PHC1506), human EGF recombinant protein (PHG0311L), Dulbecco´s modified Eagle´s medium (DMEM), Glutamax™ Supplement, pyruvate (10569010), fetal bovine serum (FBS) (A5256701), Mammalian protein extraction reagent (M-PER 78501), Halt protease, phosphatase inhibitor cocktail, and Tween™ 20 Surfact-Amps™ detergent solution, EcoR1 (ER0271), and BShT1 (Age1) (ER1461) were obtained from ThermoFisher Scientific, United States; Vitronectin XF™ (07180), human/mouse recombinant activin A (78132), mTeSR1™ medium (85850) were obtained from StemCell Technologies™ (Canada); CHIR99021 (SML1046), L-Ascrobic acid (A4544), dorsomorphin (P5499), nicotinamide (N0636), retinoic acid (R2625), monothioglycerol (MTG6145), and penicillin/streptomycin (P4333) were obtained from Sigma-Aldrich, United States; SANT-1 (559303-5MG), Immobilon-P PVDF membrane (IPVH00010), and Luminata Crescendo Western HRP Substrate, chloroquine diphosphate salt (C6628), calcium chloride (C5670), HEPES Buffer (H4034), polybrene (TR-1003-G), and doxycycline hyclate (D5207) were obtained from Merck Millipore, Darmstadt, Germany; Bicinchoninic acid kit (BCA) (786-570) was acquired from GBioscience United States; Laemmli sample buffer 2X (161-0737) was acquired from Bio-Rad, United States; SURE2 Supercompetent cells (200152) were obtained from Agilent, United States; Ampicillin, sodium salt, irradiated (11593027), carbenicillin disodium salt (10177012), and puromycin dihydrochloride (A1113808) were obtained from Gibco™, United States; Monofas^®^ DNA purification kit I (5010-21500) was obtained from GL Sciencies Inc, Tokyo, Japan; Lenti-X™ 293T cell line (632180) was obtained from Takara Bio, United States; Addgene-plasmid-pVSV-G (138479), addgene-plasmid-pRSV-Rev (12253), addgene-plasmid-pMDLg/pRRE (12251), and addgene-GFP shRNA tet pLKO puro (16037) were obtained from Addgene, United States; shKDM4A sequence were synthetized by Eurofins Genomic Tokyo, Japan. Primary antibodies against forkhead box A2 (FOXA2) (710730), SRY-box transcription factor 17 (SOX17) (PA5-72815), hepatocyte nuclear factor alpha (HNF4A) (PA5-82159), SRY-box transcription factor 9 (SOX9) (PA5-81966), pancreas associated transcription factor 1a (PTF1A) (PA5-112677), and Neurogenin 3 (NeuroG3) (703206),c and goat anti-rabbit IgG (H+L) horseradish peroxidase-conjugated secondary antibody (G21234), Luria Broth Base (Miller´s LB Broth Base) (1295027),

PureLink™ HiPure MaxiPrep kit (K210006), and Fluoromount-G™, with DAPI (00-4959-52) were obtained from Invitrogen, United States; Recombinant pancreatic and duodenal homeobox 1 (PDX1) (ab219207), and anti-NKX6.1 antibody (ab221549) were obtained from Abcam, United States; CoraLite^®^ Plus 488-conjugated INS Monoclonal antibody (CL488-66198), and KDM4A polyclonal antibody (29977-1-AP) were acquired from Proteintech, United States; QuickExtract™ RNA extraction kit (QER090150) was obtained from Biosearch Technologies, United States, and OneScript^®^ Plus Reverse Transcriptase (G237) was obtained from ABN (Canada).

### 2.2 Knockdown of KDM4A in iPSCs with shRNA

The KDM4A knockdown efficiency was evaluated using three shRNA plasmids (shKDM4A1, shKDM4A2, and shKDM4A3) constructed with a GFP shRNA tet pLKO puro. The plasmids were delivered into 20b iPSCs via lentiviral transduction and cultured in mTeSR1™ for 48 h. After that, the medium was changed and supplemented with doxycycline (1 µg/mL) for 48 h, and then positive clones were selected using puromycin (1µg/mL) for another 48 h. The KDM4A expression was quantified by real-time PCR. Based on the mRNA expression, shKDM4A3 (**Table 1**) showed the highest inhibition effect of KDM4A (data not shown).

**Table 1:** shKDM4A used to knock down (shKDM4A3).

Name	Forward	Reverse
shKDM4A3	CCGGGACTGCTGTTTATGCTCATTACTCGAGTAATGAGCATAAACAGCAGTCTTTTTG	AATTCAAAAAGACTGCTGTTTATGCTCATTACTCGAGTAATGAGCATAAACAGCAGTC

### 2.3 iPSCs differentiation

Two differentiation assays were performed on pancreatic cells during the present study. The first one was performed according to the methodology previously published (27). The second one followed the same method with the variation that the differentiation medium was always supplemented with doxycycline at a concentration of (1 µg/mL) to guarantee the expression of our shKDM4A3 inserted in the cells during differentiation experiments.

### 2.4 RT-PCR and qPCR analysis of gene expression

RT-PCR and qPCR were performed as previously described (27). Total RNA was isolated using QuickExtract™ RNA extraction kit and then reverse transcribed into cDNA using OneScript^®^ Plus Reverse Transcriptase following the manufacturer´s instruction. Quantitative PCR was performed on a CFX Opus 96 Real-Time PCR System (Bio-Rad, United States) by using Luna^®^ Universal qPCR Master Mix (M3000S) (New England Biolabs, United States). The primer list used for qPCR is shown in **Table 2**. The amplification conditions were as follows: Denaturation at 95°C for 1 minute, followed by 40 cycles of denaturation at 95°C for 15 seconds, annealing at 60°C for 30 seconds, and extension at 72°C for 30 seconds. Expression data were normalized relative to the B-Actin transcript level. The fold change for each gene was calculated using the 2^^-ΔΔCt^ method.

**Table 2:** Primer list for qPCR.

Name	Forward		Reverse
Kdm4a	TGCGGCAAGTTGAGGATGGTCT	GCTGCTTGTTCTTCCTCCTCATC	
foxA2	GGAACACCACTACGCCTTCAAC	AGTGCATCACCTGTTCGTAGGC	
sox17	ACGCTTTCATGGTGTGGGCTAAG	GTCAGCGCCTTCCACGACTTG	
hnf4A	GGTGTCCATACGCATCCTTGAC	AGCCGCTTGATCTTCCCTGGAT	
pdx1	GAAGTCTACCAAAGCTCACGCG	GGAACTCCTTCTCCAGCTCTAG	
sox9	AGGAAGCTCGCGGACCAGTAC	GGTGGTCCTTCTTGTGCTGCAC	
ptf1A	GAAGGTCATCATCTGCCATCGG	CCTTGAGTTGTTTTTCATCAGTC	
nkx6.1	CCTATTCGTTGGGGATGACAGAG	TCTGTCTCCGAGTCCTGCTTCT	
ngn3	CCTAAGAGCGAGTTGGCACTGA	AGTGCCGAGTTGAGGTTGTGCA	
ins	ACGAGGCTTCTTCTACACACCC	TCCACAATGCCACGCTTCTGCA	
gcg	CGTTCCCTTCAAGACACAGAGG	ACGCCTGGAGTCCAGATACTTG	
sst	CCAGACTCCGTCAGTTTCTGCA	TTCCAGGGCATCATTCTCCGTC	
actB	CACCATTGGCAATGAGCGGTTC	AGGTCTTTGCGGATGTCCACGT	

### 2.5 Immunofluorescence

After differentiation experiments, the cells were washed twice with DPBS buffer, followed by the addition of Krebs-Ringer buffer (KRB) with 20 mM glucose to induce insulin expression. The cells were fixed in 2% paraformaldehyde for 10 minutes, washed twice with 1X PBS, permeabilized with 0.3% Triton X-100 for 10 minutes, washed twice with 1X PBS for 5 minutes, and then blocked with 2% BSA for 40 minutes. Then, they were incubated overnight in a wet chamber with a primary antibody (CoraLite^®^ Plus 488-conjugated INS Monoclonal antibody). Finally, the cells were washed twice with 1X PBS for 5 minutes, and Fluoromount-G™, with DAPI (00-4959-52), was added to stain the nuclei at room temperature until the Fluoromount-G™ had dried. Images were captured using a fluorescent microscope.

### 2.6 Western blot

The western blot technique was performed as previously described (27) to determine the expression of characteristic proteins during pancreatic differentiation. Likewise, we determined the inhibition of KDM4A expression during each stage of differentiation following the same method.

### 2.7 Glucose-stimulated insulin secretion

According to manufacturer instructions, insulin expression was determined using the Abcam Human Insulin ELISA kit (AB100578), United States. Briefly, Pancreatic beta-like cells (PβLC), Pancreatic beta-like cells KDM4A-KD (PβLC-KD), and INS 823/13 cell line were washed two times with DPBS buffer followed by an incubation with Krebs-Ringer buffer with 2.5 mM of glucose at 37°C for 1 hour. Next, the cells were washed twice with DPBS, followed by incubation in Krebs-Ringer buffer with 20 mM glucose at 37°C for 1 hour. Supernatants from low- and high-glucose samples were collected, and insulin levels were measured in triplicate in each case.

### 2.8 Statistical analysis

All experiments were conducted at least three times. Data were displayed as mean ± SD. A two-tailed, unpaired Student´s t-test was performed to assess statistical significance, and p < 0.05 was considered significant.

## 3 Results

### 3.1 Effect of shKDM4A on iPSCs self-renewal and growth rate

**Figure 1:**
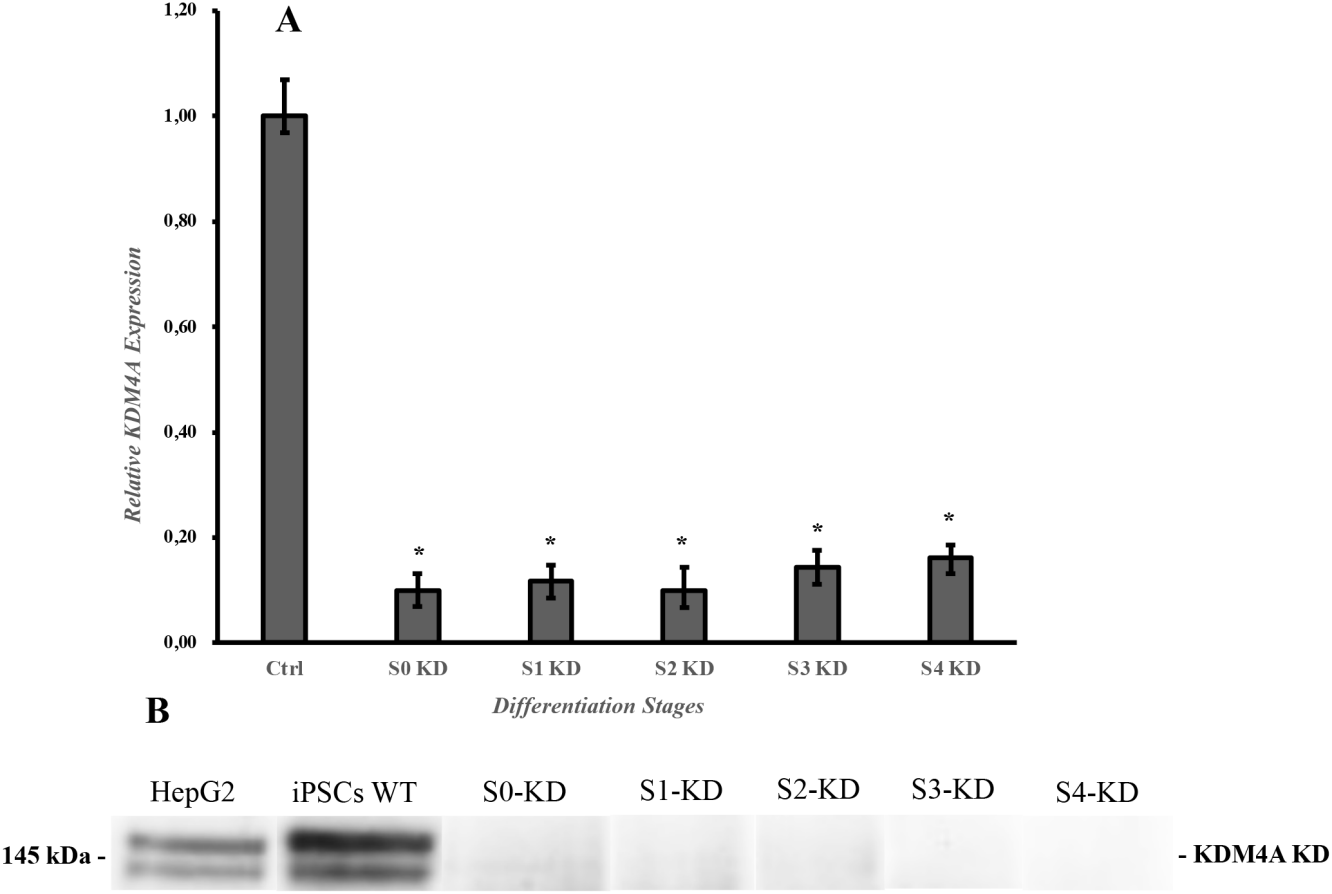
Expression of the KDM4A protein after lentiviral infection. **(A)** qPCR results of the knockdown of the protein. Expression data are normalized to the Ctrl (b-actin, transcript level). Each experiment was performed in triplicate. Bars under the same symbol (*) are statistically different under the two-tailed, unpaired Student´s t-test compared to the Ctrl expression level. *p < 0.05. **(B)** KDM4A protein knockdown during the differentiation protocol. Proteins were extracted at the end of each differentiation stage and detected using specific antibodies against KDM4A. HepG2 and iPSCs WT (wild type) were used as controls. S0-KD, Stage 0 knockdown; S1-KD, Stage 1 knockdown; S2-KD, Stage 2 knockdown; S3-KD, Stage 3 knockdown; S4-KD, Stage 4 knockdown; KDM4A-KD, Histone lysine demethylase 4A knockdown.

We found that the shKDM4A-tet-puro-pKLO reduced the KDM4A expression in the iPSCs infected with the lentiviral particles. Quantitative PCR and western blot results (**Figures 1A, B**) showed an inhibition in the protein and gene expression in all the experiments compared to untreated cells used as controls (HepG2 cell line and iPSCs). Also, the growth and expansion rate of the iPSCs infected with the shKDM4A was monitored during all the experiments to guarantee enough cell confluency to perform further experiments. We found that iPSCs-KDM4A-knockdown (iPSCs-KD) had a lower growth and expansion rate than iPSCs without shKDM4A infection (wild type) (data not shown). The results indicate that KDM4A plays a key role in the iPSC self-renewal and growth rate by influencing cell proliferation and cycle arrest (16).

### 3.2 Effect of shKDM4A on iPSCs differentiation to pancreatic ß-like cells

Two different differentiation assays were performed following the methodology previously reported (27, 28). During the first differentiation assay, iPSCs were infected with our shKDM4A by transfection with lentivirus particles to determine the effect that KDM4A knockdown would have during the differentiation process. During all the experiments, KDM4A inhibition was ensured through medium supplementation using doxycycline 1 µg/mL.

**Figure 2:**
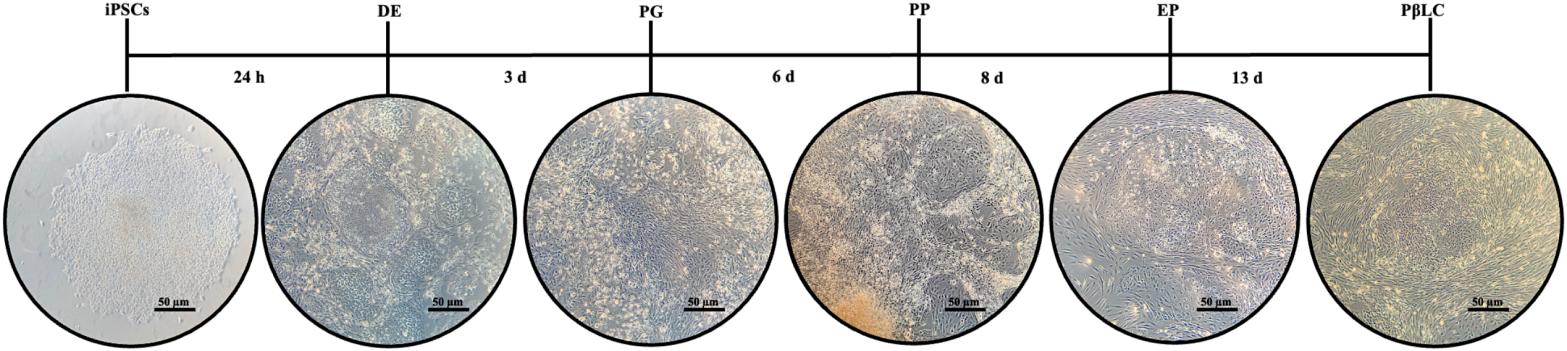
Schematic representation of the differentiation process of the iPSCs KDM4A knockdown (iPSCs-KD) to pancreatic β-like cells (PβLCs). iPSCs were cultured in a 12-well plate coated with vitronectin XF™ in a sequential protocol for 13 days. The mTeSR1™ was supplemented with different cofactors and small molecules. Images were captured with a phase contrast microscope (ZEISS AX10). DE, Definitive endoderm; PG, Pancreatic gut tube; PP, Pancreatic progenitor; EP, Endocrine progenitor; PβLC, Pancreatic beta-like cell; d, days. Black bar 50 µm.

It is essential to highlight that although the iPSC colonies maintained their typical growth characteristics before the infection (defined borders, scarce cytoplasm, large nuclei, and aggregates growth), these morphological growth characteristics disappeared during the first 72 hours of experiments (**Figure 2**). Likewise, the growth rate and cell density were affected due to the knockdown of KDM4A, demonstrating that inhibition of the expression of this protein can influence the maintenance of pluripotency of iPSCs, as well as directly influence the morphological characteristics of the cells (15).

**Figure 3:**
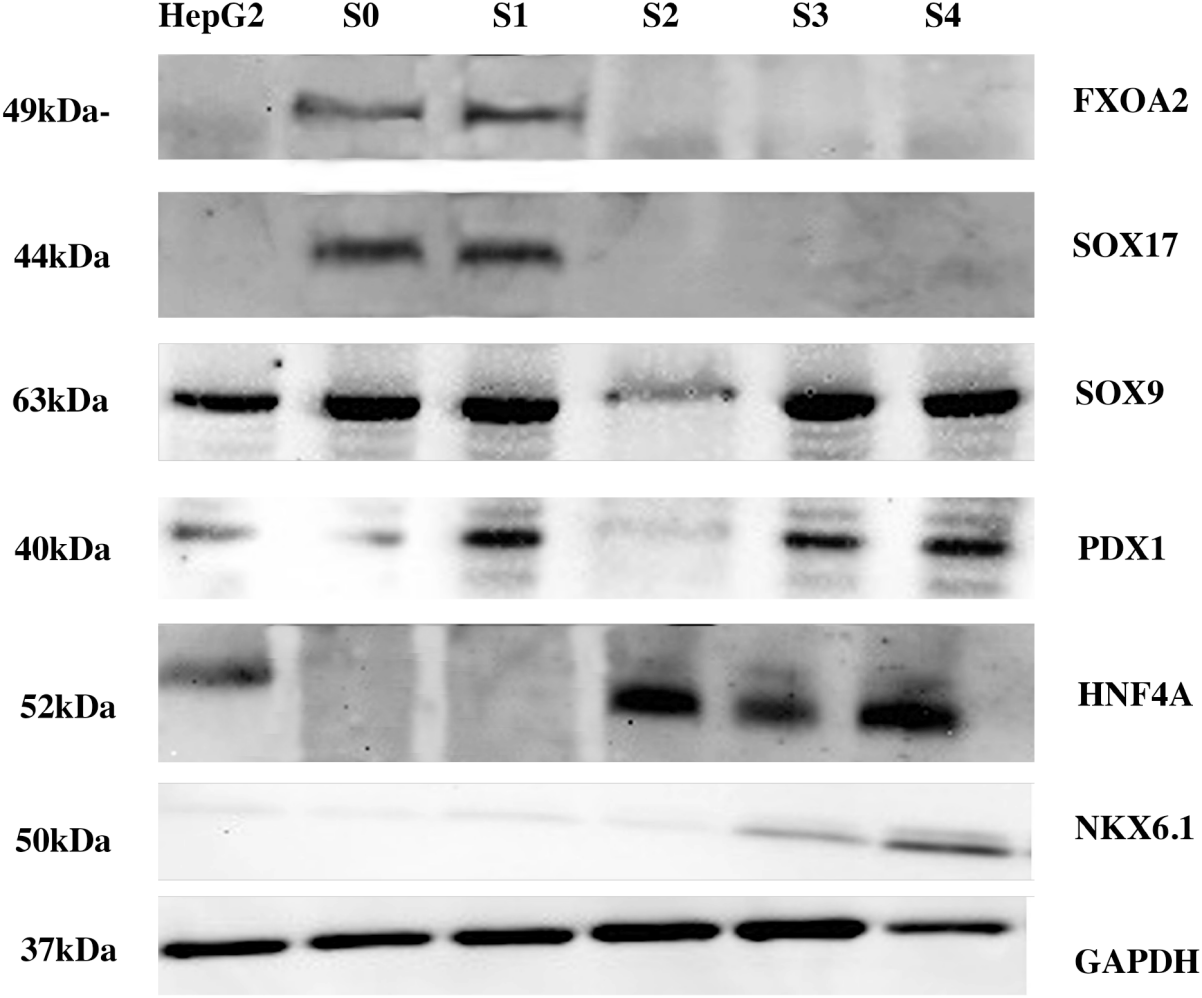
Western blot result of the differentiation process. The figure represents all the proteins detected during different differentiation stages of the iPSCs KDM4A-KD. Proteins are detected individually at each differentiation stage. A HepG2 cell line was used as the control for the experiment. The approximate molecular weight of the protein (kDa) is shown. SOX17–44 kDa; FOXA2–49 kDa; HNF4A 52 kDa; PDX1–40 kDa; SOX9–63 kDa; PTF1A 42 kDa; NKX6.1–50 kDa. S0, Stage 0; S1, Stage 1; S2, Stage 2; S3, Stage 3; S4, Stage 4.

**Figure 4:**
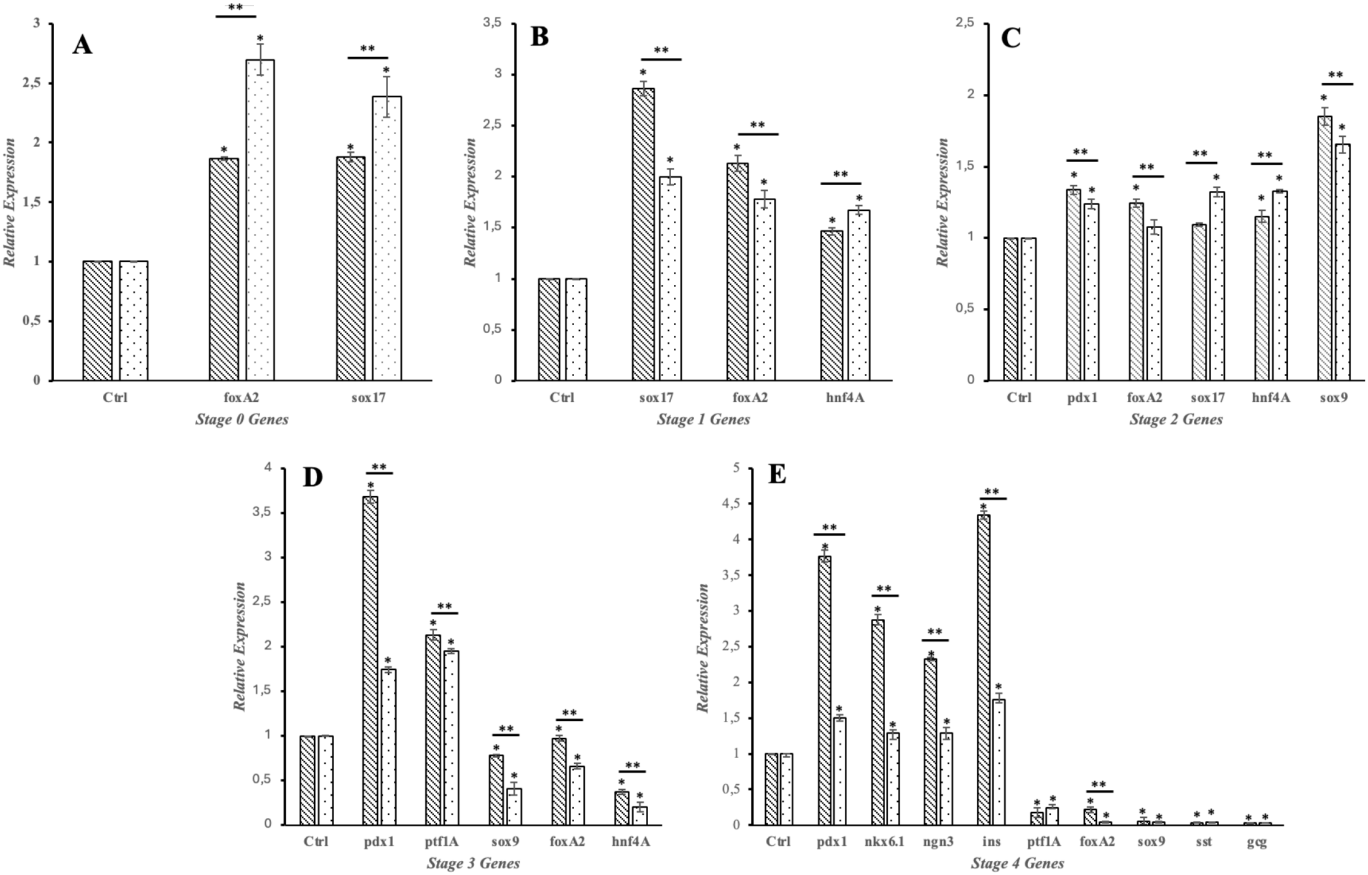
Gene expression during differentiation. Line bars represent gene expression in WT iPSCs throughout the differentiation protocol, while dotted bars represent gene expression in KDM4A-KD iPSCs. Expression levels were normalized to the control (β-actin transcript). Each experiment was performed in triplicate. Bars marked with the same symbol (*) indicate a statistically significant difference compared to the control group (two-tailed, unpaired Student’s t-test, *p < 0.05). Bars marked with the same symbol (**) indicate a statistically significant difference between the two groups (WT and KDM4A-KD, two-tailed, unpaired Student’s t-test, **p < 0.05). **(A)** Stage 0 (DE); **(B)** Stage 1 Gene expression (PG); **(C)** Stage 2 Gene expression (PP); **(D)** Stage 3 Gene expression (EP); **(E)** Stage 4 Gene expression (PβLC). foxA2, Forkhead box A2; sox17, SRY-box transcription factor 17; hnf4A, Hepatocyte nuclear factor 4 alpha; pdx1, Pancreatic and duodenal homeobox 1; sox9, SRY-box transcription factor 9; nkx6.1, Nk6 homeobox 1 protein; ngn 3, Neurogenin-3; ins, Insulin; ptf1A, Pancreas associated transcription factor 1A; sst, Somatostatin; cgc, Glucagon.

To further examine the effect of KDM4A protein knockdown on the process of pancreatic differentiation of iPSCs to pancreatic β cells, western blot (**Figure 3**) and qRT-PCR (**Figure 4**) assays were performed to evaluate the expression behavior of characteristic proteins and genes associated with the process.

The results shown in **Figure 3** indicate that proteins involved in endodermal differentiation were expressed at each stage of the protocol. However, when comparing gene expression between wild-type iPSCs (iPSCs-WT) and KDM4A knockdown iPSCs (KDM4A-KD), both differentiated under the same conditions, we observed a significant reduction in gene expression related to the differentiation process in KDM4A-KD cells (**Figure 4**, dotted bars) compared to WT cells (**Figure 4**, line bars).

These findings suggest that knocking down KDM4A, a protein involved in histone demethylation, directly affects cell differentiation *in vitro*. The slower cell growth and reduced expression of pancreatic differentiation genes observed may indicate that proper epigenetic regulation, facilitated by KDM4A, is essential for successful differentiation (29, 30).

The differences in gene expression between WT iPSCs and KDM4A-KD iPSCs are shown in **Figure 4**. Notably, throughout the assay, gene expression shown by KDM4A-KD iPSCs (dotted bar) was lower than that shown by WT iPSCs (line bar). However, the expression of proteins associated with the differentiation process (**Figure 3**) did not show an absence in the expression of any of the characteristic proteins, which is why it was considered that inhibiting the expression of the KDM4A protein would not influence pancreatic differentiation. However, when both cell groups obtained after 14 days of the protocol were analyzed (**Figure 4E**), we noticed the main differences at the gene expression level.

Notably, the most relevant differences in gene expression were observed at the expression level of crucial genetic markers of functional pancreatic β cells, such as pdx1, ngn3, and ins, which could indicate that the cells have not entirely differentiated into pancreatic β cells (31). Likewise, when we examined the expression of essential genes at the pancreatic β cell maturation level, we found that differentiated KDM4A-KD iPSCs presented low levels of nkx6.1 expression compared to WT iPSCs.

The reduced expression of key pancreatic genes observed in PβLCs derived from KDM4A-KD iPSCs suggests that the knockdown of KDM4A disrupts the proper differentiation process. Although these cells express endodermal differentiation proteins (**Figure 3**) and pancreatic differentiation genes (**Figure 4**), their low gene expression may indicate inefficiencies in the protocol, cellular immaturity, or functional limitations, which could compromise their use in regenerative medicine (32). Notably, the low expression of the critical pancreatic β-cell lineage marker *ngn3* during the final differentiation stage (Stage 4) underscores this issue (33, 34). These findings suggest that KDM4A knockdown may promote hypermethylation processes, impairing lineage specification, particularly for pancreatic β-cell differentiation, and ultimately restricting their functionality and development (35).

### 3.3 PβLC KDM4A-KD insulin secretion

**Figure 5:**
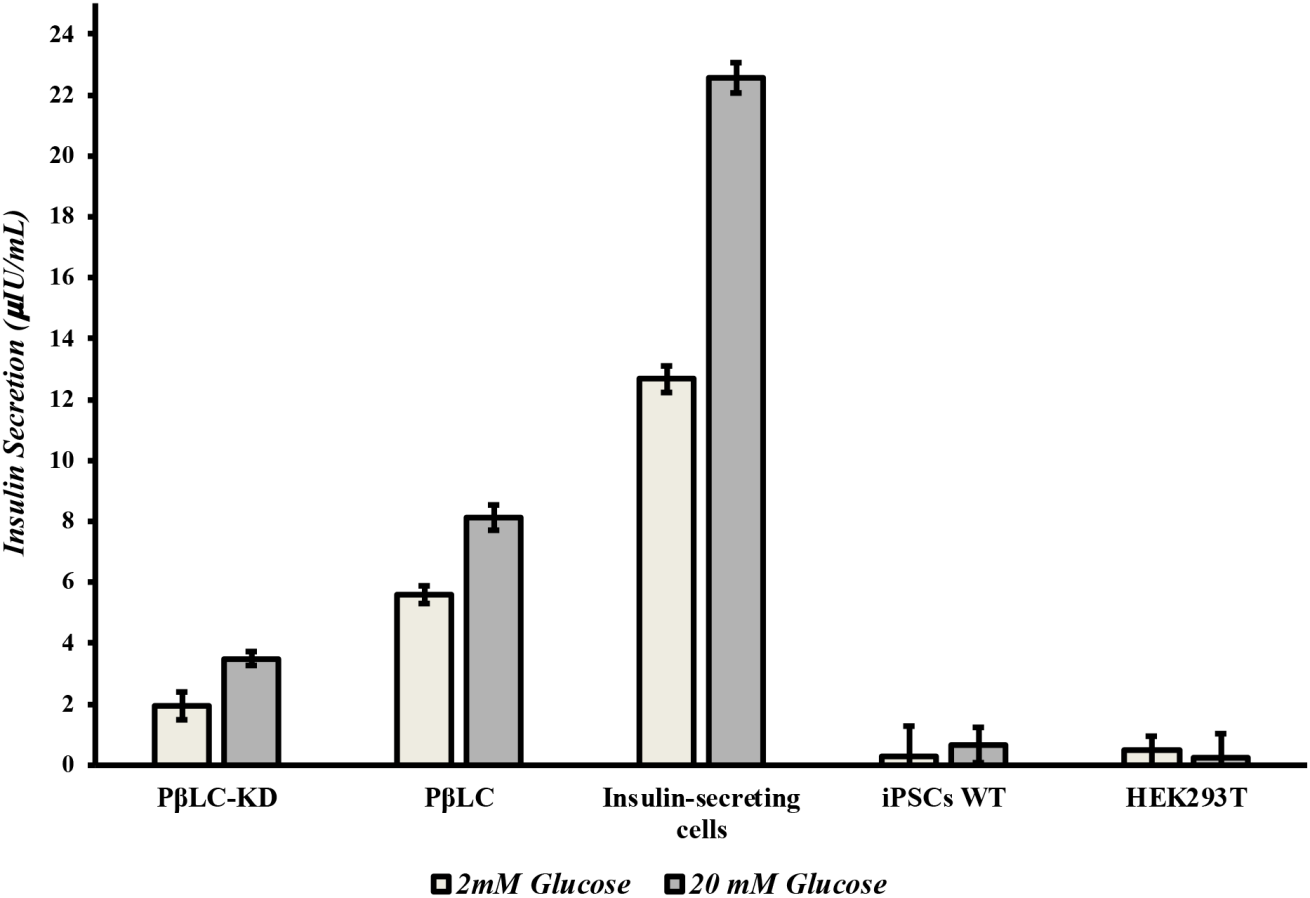
ELISA measurements of human insulin. Basal insulin was measured one-hour post-treatment with 2 mM glucose (close Barr). Stimulated insulin was measured one-hour post-treatment with 20 mM glucose (open Barr). All measurements were performed by triplicate. PβLC-KD, pancreatic β-like cells KDM4A-KD; PβLC, pancreatic β-like cells; Insulin-secreting cells (INS823/13); iPSC WT, Induced pluripotent cells without differentiation; HEK293T, Human embryonic kidney cells.

To evaluate the functionality of KDM4A-KD pancreatic β-like cells (PβLC-KD), glucose-stimulated insulin secretion (GSIS) and immunofluorescence (IF) assays were performed to assess the impact of KDM4A knockdown on their functional capacity. Insulin secretion was measured using a glucose challenge at two concentrations: low (2 mM) for basal insulin levels and high (20 mM) for stimulated secretion, with an ELISA test quantifying insulin level (**Figure 5**). The results revealed that while PβLC-KD cells responded to glucose, their insulin secretion levels were significantly lower (1.94 and 3.49 µIU/mL) compared to PβLC-WT (5.59 and 8.12 µIU/mL) and INS 823/13 cells (12.67 and 22.56 µIU/mL). This reduced functionality may be attributed to the low expression of key pancreatic β-cell genes, including *pdx1*, *ngn3*, and *nkx6.1* (**Figure 4E**). These are critical for proper cell function and were negatively impacted by the differentiation methodology used in this assay (36).

**Figure 6:**
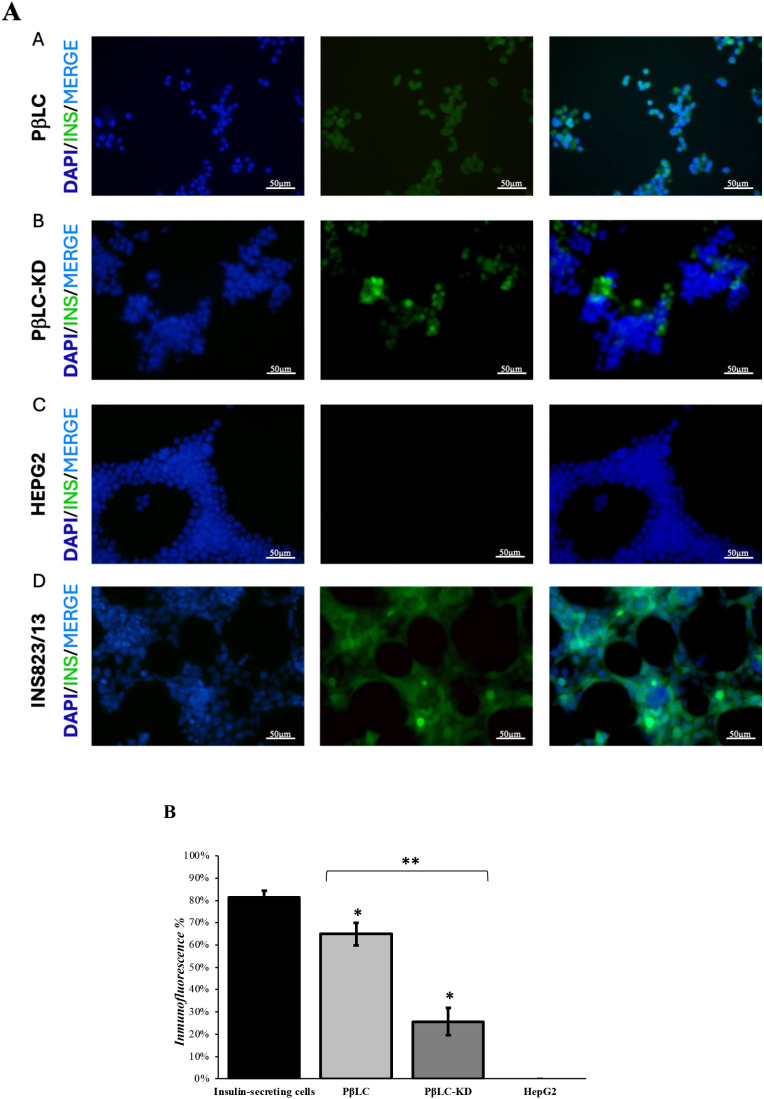
**(A)** Immunofluorescence staining showing insulin (green), DAPI (blue), and merge pictures. The results showed that PβLC and PβLC-KD express insulin in response to glucose. However, insulin production in response to glucose by PβLC-KD was lower than that of PβLC, which would be consistent with the results obtained by qRT-PCR. Scale bar = 50 µm. All images were obtained on an Eclipse Ni-E microscope (Nikon) and analyzed with ImageJ software (National Institutes of Health) at 80 ms of exposure. **(B)** Insulin expression percentage. The fluorescence expressed by insulin was normalized to that expressed by the nucleus. Bars under the same symbol (*) are statistically different under the two-tailed, unpaired Student’s t-test (p < 0.05*; p < 0.05** n = 3). A. PβLC: Pancreatic β-like cells; B. PβLC-KD: Pancreatic β-like cells KDM4A-KD. C. HepG2: Hepatoblastoma cell line (negative control). D. INS823/13: Rat insulinoma cell line (insulin-secreting cells).

We performed an immunofluorescence assay comparing PβLC-KD, PβLC, and insulin-producing cells to evaluate insulin expression and functionality. The results, shown in **Figure 6**, were obtained after exposing the differentiated cells (PβLC and PβLC-KD) and insulin-producing cells to 20 mM glucose for 1 hour, followed by insulin detection using specific antibodies. As depicted, INS823/13 cells (**Figure 6D**) exhibited the highest insulin production, followed by PβLC (**Figure 6A**) and PβLC-KD (**Figure 6B**). HepG2 cells served as a negative control for the assay.

The low insulin expression observed in PβLC-KD cells may be linked to changes in methylation patterns in the promoters of key pancreatic β-cell identity genes, such as *pdx1*, *nkx6.1*, and *ngn3* (31, 37). Previous studies have suggested that hypermethylation of these promoter sequences can contribute to the development of type 2 diabetes mellitus (T2DM), highlighting the possibility that knockdown of proteins involved in histone demethylation could directly impair the production of functional insulin-producing cells, as evidenced in **Figures 4**–**6** of this study (38, 39).

Moreover, the reduced expression of functional pancreatic β-cell genes (*pdx1*, *nkx6.1*, and *ngn3*; **Figure 4E**, dotted bar), the lower insulin secretion in response to glucose (**Figure 5**, PβLC-KD), and the diminished fluorescence in the immunofluorescence assay (**Figure 6B**) strongly suggest deficiencies in the pancreatic differentiation protocol. These results, when compared to PβLC-WT cells, provide clear indicators of the challenges associated with KDM4A knockdown during differentiation. (40–42).

These findings collectively demonstrate that the knockdown of the KDM4A protein negatively impacts the generation of functional pancreatic β-like cells capable of producing insulin in response to glucose and hinders the maturation of the differentiated cells.

## 4 Discussion

The present study provides novel evidence that KDM4A knockdown significantly impairs the differentiation of iPSCs into pancreatic β-like cells. Silencing KDM4A reduced the expression of key transcription factors involved in β-cell identity and markedly diminished both insulin content and glucose-stimulated insulin secretion (GSIS). These findings support the role of KDM4A as a critical epigenetic modulator in β-cell fate specification, suggesting that histone demethylation is a required step for the acquisition of functional maturity during pancreatic endocrine differentiation.

Our data support the hypothesis that KDM4A regulates β-cell differentiation by removing H3K9me3, a repressive histone mark associated with chromatin condensation and transcriptional silencing. The reduction in β-cell marker expression and insulin output observed in KDM4A-KD cells suggests that this demethylase facilitates chromatin accessibility at endocrine regulatory loci. These results are consistent with the idea that unresolved repressive chromatin states may act as developmental roadblocks, limiting transcriptional engagement of β-cell gene networks (43).

The differentiation defects observed in KDM4A-deficient cells align with prior studies reporting incomplete β-cell maturation from pluripotent stem cells, often yielding polyhormonal or immature cells (18, 19, 44), Kaestner and colleagues have highlighted that the persistence of bivalent or repressive chromatin domains—notably H3K9me3 and H3K27me3—can prevent full endocrine specification (6, 45). Additionally, work in primate SCNT models has demonstrated that KDM4-mediated removal of H3K9me3 is essential for unlocking transcriptional plasticity and supporting embryonic development, thereby reinforcing the notion that H3K9me3 demethylation is a prerequisite for lineage conversion (22, 26).

Epigenetic control is increasingly recognized as a primary regulatory layer in determining pancreatic lineage. Beyond transcription factor binding, chromatin accessibility shaped by histone modifications defines which genes can be activated during endocrine differentiation (46, 47). The present findings suggest that KDM4A may function as a gatekeeper of β-cell identity, ensuring that lineage-specific genes escape heterochromatic repression at key developmental transitions. Moreover, consistent with its role in other biological systems, reduced KDM4A expression may stabilize repressive chromatin structures, thereby altering both transcriptional programs and cellular identity (48, 49).

Knockdown of KDM4A resulted in downregulation of core endocrine transcription factors—including *PDX1*, *NKX6.1*, *INS*, and *NGN3*—during late stages of differentiation. This impairment was not due to failure in general differentiation capacity, as insulin expression was detectable, but rather reflected a disrupted specification program incompatible with full β-cell identity. Our findings contrast with prior studies suggesting that general KDM inhibition enhances endodermal gene expression and instead underscore the lineage-specific requirement of KDM4A in β-cell commitment (50, 51).

While this study focused on β-cell identity and function, future work should address whether KDM4A knockdown diverts cells toward alternate pancreatic fates, such as exocrine or polyhormonal lineages. Planned CUT&Tag assays targeting H3K9me3 will provide genome-wide insight into how KDM4A modulates chromatin during β-cell specification. In addition, single-cell transcriptomic approaches and lineage-tracing experiments will be instrumental in elucidating fate trajectories. These strategies will help establish whether KDM4A activity not only enables β-cell differentiation but also prevents off-target lineage activation, with direct implications for improving stem cell-based therapies for diabetes.

Taken together, our findings establish KDM4A as a key chromatin-modifying enzyme required for endocrine lineage commitment. By clarifying its role in the epigenetic regulation of β-cell development, this study contributes to the refinement of differentiation protocols and supports the broader application of epigenetic editing strategies in regenerative medicine. Future experiments focusing on KDM4A overexpression are essential to determine whether it could enhance differentiation protocols and improve the yield of functional pancreatic β-cells with a higher maturation capacity. If knockdown experiments revealed specific repression effects during differentiation, it is plausible that KDM4A overexpression may help optimize laboratory methodologies for generating pancreatic β-cells, potentially advancing regenerative therapies for diabetes mellitus.

## Data Availability

The original contributions presented in the study are included in the article/**Supplementary Material**. Further inquiries can be directed to the corresponding author/s.

## Supplementary material

Supplementary Figure 1 Western blot showing KDM4A knockdown (KDM4A-KD) versus control iPSCs (WT) and HepG2 cells (hepatic cells). Time-course of KDM4A protein across differentiation stages (S0–S4; S0, undifferentiated iPSC; S1, definitive endoderm; S2, pancreatic progenitor; S3, endocrine progenitor; S4, β-like cells).

Supplementary Figure 2 Immunofluorescence for H3K9me3 (green) with DAPI (blue) in control iPS. s (top) and KDM4A-KD iPSCs. Anti-trimethyl H3K9 (Abcam ab10812) antibodies at 1:250 dilution, followed by three washes with PBS containing 0.01% Triton X-100 and then incubation with Alexa Fluor 488 (1:500 dilution, Abcam 150077).

Supplementary Figure 3 Side-by-side comparison of PbLC vs PbLC-KD for key β-cell markers. **(A)** Western blots comparing PbLC and PbLC-KD for NKX6.1 and PDX1, with GAPDH as loading control. **(B)** Normalized densitometry (GAPDH-normalized; mean ± SEM, n = 3, two-tailed unpaired test (or mixed-effects model if blocking by differentiation batch; see Methods); p values reported. This panel illustrates the impact of KDM4A knockdown on two hallmark β-cell specification proteins
