# Supplementary figures and images for: Deciphering the epigenetic role of KDM4A in pancreatic β-like cell differentiation from iPSCs
Source: Front Endocrinol (Lausanne). 2025 Oct 31;16:1697097. doi: 10.3389/fendo.2025.1697097 (PMC12615240; doi:10.3389/fendo.2025.1697097)

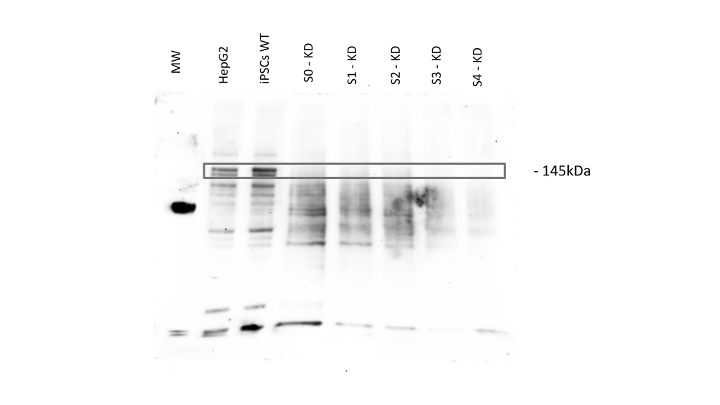

Supplement: Supplementary Figure 1 [file Image1.tiff]

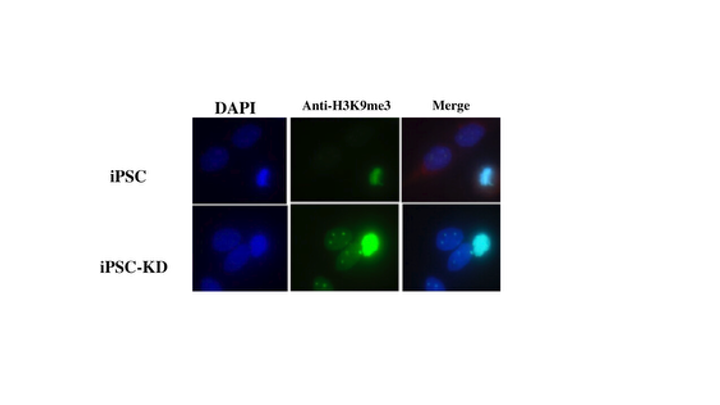

Supplement: Supplementary Figure 2 [file Image2.tiff]

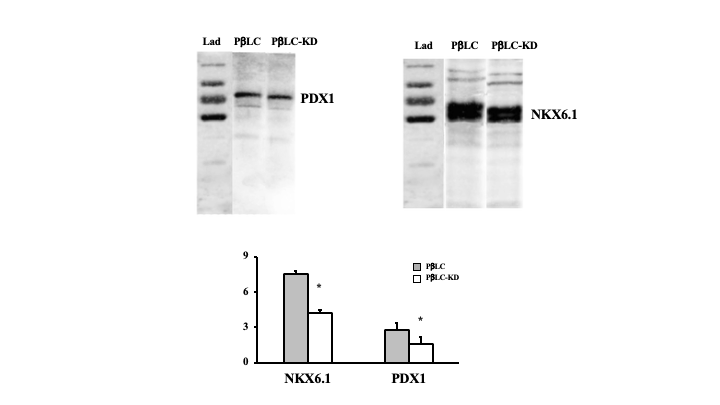

Supplement: Supplementary Figure 3 [file Image3.tiff]
